# Pulp–Dentin Tissue Healing Response: A Discussion of Current Biomedical Approaches

**DOI:** 10.3390/jcm9020434

**Published:** 2020-02-05

**Authors:** Dishant Shah, Tyler Lynd, Donald Ho, Jun Chen, Jeremy Vines, Hwi-Dong Jung, Ji-Hun Kim, Ping Zhang, Hui Wu, Ho-Wook Jun, Kyounga Cheon

**Affiliations:** 1 Department of Biomedical Engineering, University of Alabama at Birmingham, 1825 University Blvd, Birmingham, AL 35294, USA; Dshah9542@gmail.com (D.S.); tlynd@uab.edu (T.L.); donaldho@uab.edu (D.H.); cj1016@uab.edu (J.C.); jbvines@gmail.com (J.V.); hwjun@uab.edu (H.-W.J.); 2 Department of Oral & Maxillofacial Surgery College of Dentistry, Yonsei University, 50-1 Yonsei-Ro, Seodeamun-Gu, Seoul 03722, Korea; cancer7@yuhs.ac; 3 Department of Dentistry, Wonju College of Medicine, Yonsei University, 20 Il-San-ro, Wonju, Gangwon-Do 26426, Korea; pedo@yonsei.ac.kr; 4 Department of Pediatric Dentistry, University of Alabama at Birmingham, 1919 7th Avenue S, Birmingham, AL 35294, USA; pingz@uab.edu (P.Z.); hwu@uab.edu (H.W.)

**Keywords:** pulp–dentin tissue, inflammatory response, immune cells, mesenchymal stem cells, chemical signaling, biomaterials, tissue regeneration, nitric oxide

## Abstract

Dental pulp tissue exposed to mechanical trauma or cariogenic process results in root canal and/or periapical infections, and conventionally treated with root canal procedures. The more recent regenerative endodontic procedure intends to achieve effective root canal disinfection and adequate pulp–dentin tissue regeneration; however, numerous limitations are reported. Because tooth is composed of vital soft pulp enclosed by the mineralized hard tissue in a highly organized structure, complete pulp–dentin tissue regeneration has been challenging to achieve. In consideration of the limitations and unique dental anatomy, it is important to understand the healing and repair processes through inflammatory-proliferative-remodeling phase transformations of pulp–dentin tissue. Upon cause by infectious and mechanical stimuli, the innate defense mechanism is initiated by resident pulp cells including immune cells through chemical signaling. After the expansion of infection and damage to resident pulp–dentin cells, consequent chemical signaling induces pluripotent mesenchymal stem cells (MSCs) to migrate to the injury site to perform the tissue regeneration process. Additionally, innovative biomaterials are necessary to facilitate the immune response and pulp–dentin tissue regeneration roles of MSCs. This review highlights current approaches of pulp–dentin tissue healing process and suggests potential biomedical perspective of the pulp–dentin tissue regeneration.

## 1. Introduction

**Figure 1:**
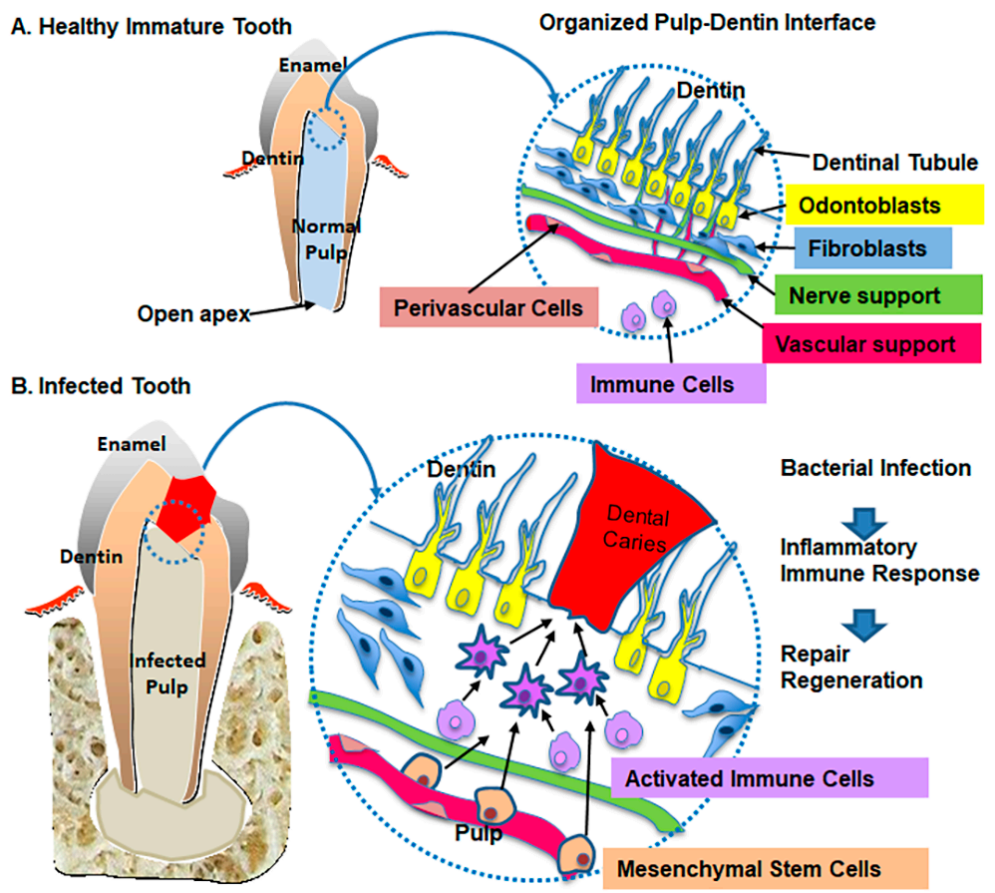
Cellular response at pulp–dentin interface: (**A**) Healthy immature tooth. (**B**) Infected tooth.

Dental infection is demonstrated as a bacteria mediated destruction of periapical tissue, including dental pulp, apex, and periodontium, accompanied by pain, swelling, mobility, and abscess formation [1]. Dental infection or periapical abscess with pulpal necrosis is the consequence of trauma; untreated/ mistreated dental caries; failed dental root canal treatment; as well as genetics, syndromes, and immune-associated medicament uses [2]. Clinically, untreated dental caries progresses to dental abscess and leads to tooth loss in 71% of adults age 45 to 64 [3]. Currently, dental abscesses of varying severity are treated using traditional root canal treatment. In young patients, tooth abscess with immature root (open apex, Figure 1A) can be treated with the regenerative endodontic procedure (REP) as one of the treatment options [4]. The REP is described as a “biologically-based procedure designed to replace damaged structures of pulp–dentin complex” using a blood clot mediated tissue healing strategy [5]. The REP aims to restore pulpal function and complete root development [6,7]. Despite some favorable clinical outcomes, the failure rate for the REP reaches 39% at 2-year follow ups [8]. Adverse results include tooth discoloration [9,10,11], root fracture [12,13,14,15], bone-like, and cementum-like periodontal tissue replacement in pulp tissues [16,17,18,19,20,21], and uncontrolled intra-canal medicament dose affecting apical stem cells [11]. Still, the revascularization capacity [22] and transplanted stem cell logistics (resources, required amount, transplantation, and immune responses) remain relevant concerns for the REP [23,24,25]. In addition, 79% of the failed treatments were attributed to persistent infection after the REP [8].

To overcome the current unsolved problems of pulp–dentin tissue regeneration, dental tissue engineering has adopted the use of various sources of stem cells, scaffold systems, and growth factors [26,27,28]. When pulp–dentin tissue is exposed to trauma or bacterial infection, regardless of the acute or chronic stages, odontoblasts recognize pathogenic signals and initiate the localized innate healing response [29,30]. The odontoblasts are positioned to enable its cell body to reach into the pulp–dentin interface and embed its long processes in the dentinal tubules as a pulp–dentin tissue defense line (Figure 1A). However, after exposure to severe bacterial penetration, there is a significant depletion of resident odontoblasts at the pulp–dentin interface, and subsequently, a release of inflammatory markers and cytokines [30]. Moreover, pulp–dentin tissue revitalization considers the aspect of wound healing involving the interactions among pulp–dentin cells (odontoblasts, mesenchymal stem cells (MSCs), immune cells, and neurovascular cells) and its chemical signaling by release of cytokines, chemokines, and other soluble factors (Figure 1B).

This review will discuss the inflammatory response on the pulp–dentin interface, which is essential for the subsequent healing and repair process. Throughout the numerous dental tissue engineering approaches, the pulp–dentin tissue interface is known to be the primary healing process. Depending on the depth of caries or bacterial penetration, the healing interface could be the line of defense and found as multiple loci from the coronal part to the root apex in any area of pulp–dentin tissue.

## 2. Characteristics of Pulp–Dentin Tissue

Normal pulp tissue is composed of organized cell layers that proliferate and differentiate to complete the root formation [31]. The pulp is mesenchymal soft tissue originating from the neural crest and extends from the central chamber to the root apex. Structurally, the pulp is loose connective tissue composed of pulp cells, collagen fibers, extracellular matrix, nerves, and blood vessels. The healthy dental pulp contains a heterogeneous mixture of cells including odontoblasts, fibroblasts, immune cells (macrophages/histiocytes, dendritic cells, and T-lymphocytes), Schwann’s cells, and vascular/perivascular cells [32]. The pulp cells are responsible for tissue repair and self-renewal along with the prepositioned inflammatory process [33]. Depending on the intensity and infiltration depth of the bacterial invasion, viable pulp cells are able to initiate the repair process with an inflammatory reaction.

During root dentin development, Hertwig’s epithelial root sheath (HERS) presents as a merged inner- and outer-enamel epithelial structure from the cervical loop of the enamel organ surrounding the root surfaces. The HERS then proliferates towards the distal end and bends at a near 45 degree angle forming an epithelial diaphragm which determines the number, shape, and size of the root [34]. HERS plays a critical role for root dentin and cementum formation in conjunction with dental papilla [35]. Progressing root development, odontoblasts produce dentin layers deposited at the pulp–dentin interface and retreat toward the root apex (apical papilla) during the early stage of root development. In the later stage of root development, the apical papilla reserves undifferentiated MSCs as a potential source of root dentin formation [36]. Along with the HERS, stem cells from apical papilla (SCAP) and follicular tissue are implicated in developing apical complex cells to establish the root and periodontium: root dentin, cementum, and the periodontal ligament [37,38].

However, after exposure to infection, the pulp–dentin interface of root structures, including radicular pulp, apical papilla, and HERS, show significant histopathologic alterations devoid of cellular components (odontoblasts and MSCs) and misplacement of cementum-like, osteo-like, periodontal ligament-like tissue [35,39,40]. The affected root canal wall is free from odontoblasts and repaired by the deposition of periodontal tissue without normal dentin structures such as odontoblasts’ processes and dentinal tubules. When the pulp tissue progresses into the necrotic phase, apical papilla demonstrate extremely reduced cellularity with a discontinuous or absent HERS [35,40].

The tooth structure is composed of inner non-mineralized pulp tissue, one directional neurovascular supply, and surrounding mineralized dentin. Due to the dental pulp being confined by mineralized tissue, the initial inflammatory/immunological reaction increases intra-canal pressure, and the pressure extends toward the root apex down the root canal, causing periapical abscess [35,41,42]. Left untreated, the abscess can cause root resorption and periodontal tissue disruption involving the periodontal ligament space and surrounding alveolar bone and result in the loss of bony support and teeth extrusion [42,43]. Therefore, the root canal treatment (endodontic therapy) intends to remove infected tissue to decrease intra-canal pressure from the dental pulp and alleviate pain [39,41]. The pulp treatment modality is decided based upon the penetration depth of the inflammation and maturity of the root apex as indicated in direct or indirect pulp capping, partial or complete pulpectomy, apexification, or apexogenesis [39,44,45]. Withstanding the pathological challenges, pulp–dentin–periodontium tissue will respond and engage in a biological defense mechanism via cell–cell communication and chemical signaling, which could function as the healing foci of repair/regeneration.

## 3. Biologic Defense Mechanism on Pulp–Dentin Tissue

Odontoblasts—dentin-producing cells—have been shown to sense bacterial pathogens at the pulp–dentin interface and respond to them by releasing proinflammatory cytokines and antibacterial agents such as nitric oxide (NO) [29,30,46]. NO is produced as part of the host defense in a nonspecific immune reaction [47]. When any pathogen bypasses the pulp–dentin interface, either by degrading the dentin or penetrating through dentin tubules, the odontoblast releases chemokines to recruit immune cells (e.g., dendritic cells (DC) and macrophages) to the infected locus [48]. Upon binding to the antigens, the immature DC will mature and influence the innate and adaptive immunity process. DCs migrate to lymph nodes where the antigens will be released to naïve CD4+ T cells, which will then differentiate to either regulatory T cells or effector CD4+ T helper cells (Th1 or Th2), depending on the cytokines present [49,50]. Th1 and Th2 influence macrophage activity and enable immune mechanisms specific to which T helper cell was produced.

Macrophages serve to phagocytose invading pathogens and can be activated by Th1 and inhibited by Th2 [51]. Macrophages are known as one of the critical mediators to influence the innate immune response [30,52]. A macrophage begins at the resting M0 state, and the cell can be polarized to a proinflammatory M1 state or an anti-inflammatory M2 state. Polarization to the M2 macrophage fosters tissue repair and inflammation resolution. The wound healing process begins with an initiated proinflammatory phase and transitions to anti-inflammatory phases, which corresponds with macrophage phenotypic alterations from M1 to M2 respectively along the release of the pro- and anti-inflammatory cytokine. The pleotropic macrophages are devoted in many stages of the wound healing process including promotion of inflammation, reparation with anti-inflammation, and synthesis of extracellular matrix (ECM) [52]. In addition, natural killer cells and natural killer T cells are other innate immune system components and are present in normal dental coronal pulp tissue as a first line of defense. Natural killer T cells support the development of Th1 and Th2 immune responses of destroying infiltrating pathogens [53]. Through the progression of dental caries and inflammation, the number of B lymphocytes are increased and associated with the modulation of DC functions [54]. Of all the leukocytes present in human dental pulp, 21% are cytotoxic CD8+ T cells, 11% are CD4+ T cells, and 4% are DCs. Importantly, the endothelium-derived NO modulates leukocyte adherence in vessel walls [55]. This characterization of the leukocyte population can give a glimpse into the initial ability of the pulp to defend itself before more leukocytes are brought towards the pulp by the circulatory system [30].

A current study reports that inflammatory responses are prerequisite and can stimulate dental tissue repair responses [56]. When the pulp is exposed to caries, bacteria, or dental filling materials, the pulp–dentin interface undergoes a mild to severe inflammatory process. The proinflammatory cytokines, tumor necrosis factor alpha (TNF-α), interferon gamma (IFN-γ), interleukin 1beta (IL-1β), IL-6, etc. are initiated to promote the host immune response, whereas the anti-inflammatory cytokines, steroids, transforming growth factor beta (TGF-β), IL-10, NO, etc. are released to limit the tissue damage. With the interactive balance between the pro- and anti-inflammatory signaling, pulp–dentin tissue can respond with cell necrosis, bone resorption, pulp calcification, or revascularization. Low levels of inflammatory cytokine signals direct the response of the cells to drive differentiation and mineralization to favor healing, whereas a high level of inflammatory cytokine signaling may result in the recruitment of more immune cells further accelerating the inflammatory response in vitro [56]. For instance, chemokine receptor 4 (CXCR4) appears to have both proinflammatory and repair processes. CXCR is critical for lymphocyte trafficking as well as hematopoiesis with organogenesis in conjunction with stromal cell derived factor-1 [57]. Nuclear Factor kappa-light-chain-enhancer of activated B cells (NF-ΚB) and p38 mitogen-activated protein kinase (MAPK) are key signaling pathways in both the proinflammatory and healing responses [58]. Notably, low levels of cytokine secretion causes mesenchymal stem cells to perform healing associated immunomodulatory and anti-inflammatory functions; however, a high level of cytokine secretion may hinder the response from the stem cells [57,59,60].

While the inflammatory phase of pulp–dentin tissue progresses, the cellular proliferation and differentiation phases are necessary for the wound healing/repair process [30,61]. During the process, vascularization is critical for the provision of nutrition and oxygen as well as the elimination of metabolic waste. Furthermore, the vascularization could guide MSCs migration from perivascular region to the inflammatory loci [62]. Having multipotential and self-renewal properties, MSCs such as dental pulp stem cells (DPSCs) differentiate into odontoblasts, fibroblasts, endothelial cells, and neural cells [63,64,65,66,67,68]. The vascularization is supported by the angiogenic signaling molecules, vascular endothelial growth factor (VEGF), basic fibroblast growth factor (bFGF), and TGF-β which are released from injured pulp cells, endothelial cells, and ECM [69,70]. Collectively, the inflammatory, proliferation, and differentiation phases collaborate to enrich a natural healing process for pulp–dentin tissue.

## 4. Mesenchymal Stem Cell Response and Pulp–Dentin Tissue Response

Stem cells possess the ability of self-renewal and pluripotency resulting in the capacity to differentiate into a variety of cell types [71]. Stem cells can be classified into three types: (1) Embryonic stem cells, which are obtained from the inner cell mass of blastocysts. Embryonic stem cells have been harvested from mice and humans; however, the actual application has been restricted due to the ethical concerns and obtaining difficulty [72]. (2) Induced pluripotent stem cells (iPSCs), which are obtained from the overexpressed growth factor treated adult cells. iPSCs are similar to embryonic stem cells at a cellular level in their ability for differentiation and self-renewal [73]. iPSCs have been harvested from fibroblasts, however, their genetic stability is still questionable [71]. (3) MSCs originate from mesoderm and possess genetic stability. Human MSCs have been isolated from a variety of sources in the human body such as bone marrow, skin, perivascular tissues, as well as the dental tissues [25,74,75,76]. Recent studies demonstrated that MSCs isolated from dental tissue may serve as a more feasible and beneficial source for the MSC-based cell therapy over bone marrow derived MSCs [77].

MSCs has been studied to perform a major role in tooth growth and repair by responding to biological stimulations and signals [78]. Depending on the depth of penetration of dental caries from dentin to pulp, an innate defense mechanism is initiated by the odontoblast, which produces a reactionary dentin layer in response to the stimulus. However, resident odontoblasts will be severely damaged by the extension of the penetration. Consequently, dental MSCs, predominantly DPSCs, reserve the capacity to migrate to the site of injury and differentiate into odontoblasts to commence reparative dentin synthesis for tissue recovery. Evidence suggests dental MSCs develop from both pericytes and differentiate into odontoblast during growth and in response to odontoblast damage [79]. DPSC differentiation is associated with the release of sequestered TGF-β following physical damage and Wnt signaling. As a result, the presence of TGF-β in dentin tubules assists in reparative dentin formation. The beta-catenin-dependent Wnt signaling pathway is stimulated upon injury and plays a fundamental role in dental MSC deployment [80]. In these studies, DPSCs have exhibited great potential to reestablish pulp-like tissue enhancing the conventional root canal treatment, which lack the ability to restore pulp vitality [78]. In addition, the immune modulatory effects of DPSCs are demonstrated by the association of immune cell proliferation and tissue inflammation suppression during the repair phase [78,81]. MSCs can promote the activation of macrophages transition from proinflammatory M1 to anti-inflammatory M2 phenotypes; therefore, MSCs are responsible for the regulation of inflammatory reactions through the secretion of cytokines, chemokines, and other factors to maintain body homeostasis and wound healing process [82,83]. By the evidenced immune-modulatory role, DPSCs are critical cells to repair the pulp–dentin tissue and restore tooth function. Therefore, genuine pulp–dentin regeneration using DPSCs is highly expected using an innovative biomedical approach in the near future.

## 5. Dental Apex and Periodontal–Tissue Response

Dental infection that progresses and reaches the periapical and periodontal interface by extended pathology as a periodontal abscess can be classified as either marginal periodontitis or apical periodontitis. Both forms of periodontitis lead to the destruction of surrounding dentin, cementum, periodontal ligament (PDL), alveolar bone, and gingival tissue [84]. During the pathologic process, the host’s innate and adaptive immune cells indirectly mediate the destruction of the periodontal tissues via inflammatory responses [85,86]. Guided tissue regeneration (GTR) following periapical surgery improves healing for the damaged and lost tissue by using autogenous cells from healthy tissue including alveolar bone, PDL, and cementum [84,87,88]. However, GTR treatments have been incapable of fostering complete and functional periodontal tissue regeneration including the proper composition of peri-apex, cementum, PDL, and alveolar bone. Rather, the treatment results in a collection of undesired fibrous connective tissue, cellular cementum, and bone tissue [84]. Although the regenerative medicine-based procedure implicates great potential, the clinical outcomes and prognoses remain in concern due to unpredictability [89]. Severity of infection and implanted biomaterials affect periapical wound healing resulting in inflammation, fibrosis, and periodontal destruction [90]. Therefore, periodontal wound healing depends on controlling and modulating the inflammatory cytokine response leading to therapeutic regeneration outcomes [91].

## 6. Nitric Oxide (NO) and Pulp–Dentin Tissue Response

Nitric oxide (NO), a short-lived radical and inert gas, reserves a vital function in regulating inflammatory response in wound repair. Innate NO is produced by immune and non-immune cells by nitric oxide synthase (NOS) via L-arginine metabolism. Among the known three isoforms, endothelial NOS (eNOS) and neuronal NOS (nNOS) are constitutively expressed and produce NO at a constant basal level. Inducible NOS (iNOS) is demonstrated using cytokines, growth factors, and inflammatory stimuli on target cells in the early phase of the inflammation and yields much greater levels of NO than the constitutive isoforms of eNOS and nNOS [92].

**Figure 2:**
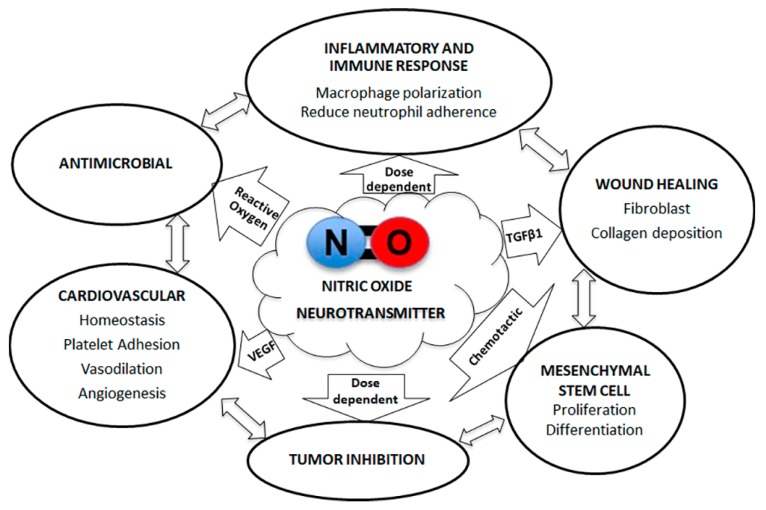
Multifunctional effects of nitric oxide for potential tissue regeneration.

As shown in the Figure 2, NO’s bioactive functions are explored and demonstrated [93]: NO is known as a neurotransmitter to mediate vasodilator tone, respiratory, genitourinary tract, and cardiac functions via activation of the cyclic guanosine monophosphate (c-GMP) [47]. NO significantly contributes to promote angiogenesis and develop mature blood vessels via recruiting perivascular and endothelial cells [94]. NO has also been shown to affect vascular endothelial growth factor (VEGF) release during angiogenesis occurring in bone remodeling [95]. Furthermore, NO reserves the ability to either inhibit the growth or directly eliminate bacteria and prevent infection. However, the molecule has an elevated risk for toxicity by direct application. Therefore, the use of biomaterials to mediate its release is important to ensure the viability of surrounding tissue [96]. NO has been implicated in the modulation of tumor biology with both tumoricidal and tumor-promoting properties. The compound can encourage tumorigenesis in cells responsive to its angiogenic and antiapoptotic roles. In contrast, NO is often cytostatic in tumors due to the suppression of DNA synthesis via the salvage pathway and the regulation of aconitase and ribonucleotide reductase. The tumor suppressing properties are augmented at elevated levels of NO [97].

Additionally, NO coordinates key processes for tissue repair at moderate levels including collagen synthesis, cell proliferation, and cellular differentiation [92]. NO derived from eNOS in bone was found to show effects on bone formation in vivo, demonstrated by compelling evidence that eNOS knockout mice exhibited large insufficiencies in bone formation [98]. A recent study reported that rat dental pulp stem cells (rDPSCs) stimulated by exogenous NO could transform into odontoblast-like cells with enhanced alkaline phosphatase (ALP) activity and expression levels of odontoblast-specific genes such as runt related factor 2 (Runx2), DMP 1, and dentin sialophosphoprotein through the NF-κB pathway, highlighting promising therapeutic possibilities for NO treatment in the clinic [99].

Furthermore, NO is known to contribute to the immune response and wound healing process by its antibacterial capacity and regulatory effect on immune cell activation and polarization [100,101,102]. NO may be associated with macrophage polarization. One study describes the role of iNOS in the suppression of M1 macrophage polarization and found NO modified tyrosine residues via nitration in Interferon regulatory factor 5, a key transcription factor for M1 macrophage polarization [101]. Likewise, an additional study confirms the effects of NO on the suppression of M1 macrophage polarization and suggests that NO encourages M2 polarization [102]. The findings highlight the immunomodulatory potential of NO to promote pulp–dentin tissue wound healing.

NO plays an important biphasic role in the function of osteoblasts and osteoclasts. Osteoblasts and osteoclasts are the two major cell types responsible for bone formation and resorption, respectively. The effect of NO on osteoblasts in vitro has been reported to be associated with the concentration of NO present in the local bone microenvironment [47]. High NO concentrations with rapid release cause proinflammatory reactions, which inhibit proliferation and differentiation and induce an apoptosis effect on osteoblasts and osteoclasts [103]. Although low NO concentrations enhanced osteoblast growth, cytokine production, and survival [104], a study demonstrates that a low concentration of NO will stimulate the Wnt/β-catenin pathway and increase osteogenic differentiation genes such as ALP, osteocalcin, collagen-1, and Runx2 [105]. An in vivo study demonstrated that a low dose of NO corresponds with an increasing number of osteoblasts, mineral apposition, and bone formation rates via angiogenesis in endochondral ossification and bone repair [105,106]. Therefore, NO releasing biomaterials may play important roles in pulp–dentin tissue regeneration.

## 7. Consideration of Potential Biomaterials for the Pulp–Dentin Tissue Revitalization

The foundation of reliable pulp–dentin tissue regeneration depends on pulp ECM tissue mimicking scaffolds, characterized as bioactive, antibacterial, and anti-inflammatory materials with a low immunogenic response and a high therapeutic drug delivery efficacy [107]. In addition, scaffolds possess biocompatibility to allow cell adhesion, proliferation, differentiation, as well as migration [108,109]. Clinically, materials for scaffolds need to be injectable, adaptable, biodegradable, and capable of gradually releasing growth factors [110]. A series of natural and synthetic polymers have been reported as a potential scaffold to substitute the soft, functional pulp tissue ECM [109,111,112]. Natural polymers provide a more natural ECM environment and limited toxicity compared to synthetic polymers. Synthetic polymers increase feasibilities of scaffold characteristics [113]. Among natural polymer scaffolds, collagen is one of the most widely used biomaterials. The natural collagen motifs give rise to its cell adhesive and biocompatible properties. Studies have shown that collagen hydrogels encourage angiogenesis, adipogenesis, and osteogenesis. Collagen hydrogels restricted mechanical property could be modified using crosslinking method; however, the attempts indicate limited success. Furthermore, batch-to-batch variation and pathogen transmission are common concerns for natural scaffolds [114,115]. Chitosan is another promising natural biomaterial for endodontic treatment. The cationic polymer has desirable biocompatibility, biodegradability, and antimicrobial capacity. Furthermore, chitosan displays potential to promote mineralization offering therapeutic potentials for both pulp and dentin tissues. However, its reduced solubility in neutral aqueous and organic solutions are current concerns and a focus of current chitosan scaffold research [114,116].

Synthetic scaffold designs provide an enhanced capacity to optimize mechanical strength and molecular delivery for a targeted therapeutic approach as shown in the Table 1 [21,143]. The table describes diverse biomedical approaches to achieve the endodontic regeneration [21,112]. Current biomaterials research pursues key functionalities for endodontic and periodontal tissue healing. For example, many novel scaffolds involve sustained delivery of therapeutic compounds including antimicrobials and signaling molecules (e.g., growth factors). Furthermore, synthetic scaffolds employ sites for enzymatic degradation to foster restoration of bio functional pulp–dentin tissue. Recently developed peptide amphiphile (PA)-based biomaterials are an example of a synthetic scaffold and offer potential promise in regenerative endodontics. The use of a PA hydrogel with stem cells from human exfoliated deciduous teeth (SHED) or DPSC in vitro demonstrated similar trends where both cell types were able to proliferate, differentiate, and yield mineralized deposits [134]. Studies implanting a self-assembled peptide scaffold containing dental stem cells reported dental pulp regeneration in one-third of the prepared dentin cylinders in rat teeth; however, full-length root pulp regeneration still needs more research [131,144].

Among the newly developed synthetic polymer, a NO releasing biomimetic nanomatrix gel was evaluated as a nature mimicking novel PA based scaffold [145]. This self-assembled PA gel mimics the ECM and provides a controlled release of NO, showing the therapeutic roles of disinfection, revascularization, and regeneration [146] (Figure 2). The antibacterial aspect of NO is critical for dental pulp regeneration to ensure a sterile pulp–dentin tissue environment and recruit healthy cell growth including DPSCs and neurovascular cells to promote revascularization. A study using the NO releasing PA gel found blood vessel formation as well as odontoblast-like cells in a beagle model; the findings draw attention to the potential of NO to promote stem cell homing and differentiation [145]. This ability to induce DPSC differentiation into dentin producing odontoblasts further highlights the potential of NO in dental pulp regeneration.

## 8. Summary

Pulp–dentin tissue regeneration is the series of healing progression from an inflammatory response, immune signaling, and cellular interaction accompanying tissue restoration upon infectious exposure. Due to the unique anatomy of the tooth, infectious tissue damage extends toward the periapical and periodontal regions; therefore, cellular communication among the pluripotent pulp stem cells and immune cells is crucial for an appropriate response. Along with cellular signaling, migrating MSCs, such as DPSCs, could proliferate and differentiate into the pulp–dentin tissue-forming cells including neurovascular tissue regrowth as part of pulp–dentin tissue healing response. The tissue-healing phase is regulated by the anti-inflammatory M2 macrophage following the inflammatory phase. In fact, MSCs are associated with the further promotion of anti-inflammatory M2 macrophage polarization. Meanwhile, consequent tissue regeneration would occur with the facilitation of ECM mimicking bioactive scaffolds and mesenchymal cellular potential. Among the numerous biomedical approaches, recent development of the NO releasing biomimetic nanomatrix gel may provide the necessary supportive function for the pulp–dentin tissue microenvironment to regenerate optimal root dentin and pulp vitality. NO possesses a significant effect on inflammation and tissue healing with the antimicrobial, angiogenic, wound healing, and immunomodulatory properties.

## Figures and Tables

**Table 1:** Summary of recent biomedical approaches in regenerative endodontics (adapted from work in [21,112]).

Stem Cells	Scaffold	Findings	Reference
Pulp fibroblasts	PGA, collagen I, alginate	Pulp-like tissue after 45 to 60 days on PGA	[117,118]
Human DPSCs	Col Type I with CP and DMP-1	New pulp-like tissue formation and organization	[119]
	Collagens I and III, chitosan, gelatin	Adhesion and proliferation	[66]
	NF-PCL/gelatin/nHA	DPSC differentiation toward an odontoblast-like cells in vitro and in vivo	[120]
	NF-PLLA	Attachment, proliferation, and differentiation of human DPSCs	[121]
	Self-assembling MDP	Pulp-like tissue formation	[111]
	DDM-PLGA/Co-Cs-HA	Potential as attractive scaffolds for odontogenic differentiation	[122]
	3D Col/HA/PLCL	DPSC Differentiation and proliferation	[123]
	Self-assembling peptide (Puramatrix^TM^)	DPSC survival, proliferation, and differentiation	[124]
	Porous chitosan/Col	Release of BMP-7 gene; DPSC differentiation into odontoblast-like cells in vitro and in vivo	[125]
Dog mobilized DPSCs	Col with G-CSF	Ectopic model, pulp-like tissue regeneration	[126]
		Complete pulp-like and dentin-like tissue regeneration	[127]
		Orthotopic model; Less volume of regenerated pulp-like tissue in aged dogs compared with that in young dogs	[128]
SHED	PLLA	Pulp-like tissue formation	[129]
	3dimension dense Col	Odontogenic cell differentiation and mineralization	[130]
	Peptide(Puramatrix^TM^) with rhCol type I	SHED injected into full-length human root canals differentiate into functional odontoblasts	[131]
DPSCs & SHED	HA/TCP	Generation of dentin or bone (SHED) and dentin-pulp-like complexes (DPSC)	[132,133]
	PA self-assembling NF	Easy to handle; introduced into small defects; cell proliferation	[134]
DPSCs & SCAPs	Poly-D,L-lactide/glycolide	Pulp-like tissue formation with vascularity and dentin-like structure	[135]
DPSCs, SCAPs, PDLSCs, and BMSSCs	PEGylated fibrin gel	All types of dental stem cells proliferated; excellent biocompatibility; insertion into small defects	[136]
No Stem Cells	Alginate with TGF-β1	Release of TGF-β1; odontoblast-like cell differentiation	[137]
	Gelatin incorporation of FGF-2	Release of FGF-2; Induces the invasion of dental pulp cells and vessels	[138]
	NF-PLGA/PLLA scaffolds with DOXY	Release of DOXY; inhibition of bacterial growth for a prolonged duration	[139]
	GF–laden peptide with VEGF, TGFβ-1, and FGF-2	Release of VEGF, TGF-β1, and FGF2; odontoblast-like cell differentiation; pulp-like tissue formation	[140]
	NF PDS II-with MET and CIP	Release MET or CIP; antimicrobial activity against *Ef* and *Pg*	[141]
	NF PDS II-HNTs	Potential in the development of a bioactive scaffold for regenerative endodontics	[142]

PGA: poly glycolic acid; DPSCs: dental pulp stem cells; Col: collagen; CP: ceramic powder; DMP-1: dentin matrix protein 1; NF: nanofibrous; PCL: poly ε-caprolactone; nHA: nano-hydroxyapatite; PLLA: Poly L-lactic acid; MDP: multidomain peptides; DDM: demineralized dentin matrix; PLGA: poly lactic-co-glycolic acid; Co–CS–HA: collagen–chondroitin sulfate–hyaluronic acid; PLCL: poly L-lactide-co-ε-caprolactone; BMP-7: human bone morphogenetic protein-7; G-CSF: granulocyte colony-stimulating factor; SHEDs: stem cells from human exfoliated deciduous teeth; rhCol: recombinant human collagen; TCP: tricalcium phosphate; PA: peptide-amphiphile; SCAPs: stem cells from root apical papilla; PDLSCs: periodontal ligament stem cells; BMSSCs: bone marrow stromal stem cells; PEG: polyethylene glycol; TGF-β1: transforming growth factor β family; FGF-2: fibroblast growth factor–2; DOXY: doxycycline; GF: growth factor; VEGF: vascular endothelial growth factor; PDS-II: nanocomposite scaffold composed of polydioxanone; MET: metronidazole; CIP: Ciprofloxacin; *Ef*: *Enterococcus faecalis*; *Pg*: *Porphyromonas gingivalis*; HNT: halloysite nanotubes. (The inserted table highlighted the current finding of recent biomedical approaches in regenerative endodontics that was adapted from the two references with the permitted citations and adaptation from the publisher (Sage Publication). I read that the policy about the table adaptation would not need mention the copyright permission).
